# Effects of veneering porcelain thickness and background shade on the shade match of zirconia-based restorations

**DOI:** 10.15171/joddd.2019.011

**Published:** 2019-04-24

**Authors:** Farhad Tabatabaian, Khotan Aflatoonian, Mahshid Namdari

**Affiliations:** ^1^Department of Prosthodontics, School of Dentistry, Shahid Beheshti University of Medical Sciences, Tehran, Iran; ^2^Private Practice, Tehran, Iran; ^3^Department of Community Oral Health, School of Dentistry, Shahid Beheshti University of Medical Sciences, Tehran, Iran

**Keywords:** Ceramic, esthetics, shade, spectrophotometry, zirconia

## Abstract

***Background***. Effects of veneering porcelain thickness and background shade on the shade match of zirconia-based restorations are unclear. The aim of this study was to evaluate the impacts of veneering porcelain thickness and background shade on the shade match of zirconia-based restorations.

***Methods***. Forty A2 shade veneered zirconia disk specimens (10 mm in diameter) were fabricated, with veneering porcelain thicknesses of 1.6, 1.8, 2.0 and 2.2 mm. Three backgrounds were made of A2 shade composite resin (A2), nickel-chromium alloy (NC) and amalgam (AM). The veneered zirconia specimens were placed on the backgrounds. CIELab values were measured with a spectrophotometer. ΔE values were measured to determine color differences between the specimens and the A2 VITA classical shade (target shade). ΔE values were compared with an acceptability threshold (ΔE=3.7). Repeated measures ANOVA, Bonferroni, and 1-sample t-test were used to analyze data (P<0.05).

***Results***. Mean ΔE values ranged between 1.9 and 5.0. The veneering porcelain thickness, the background shade and their interaction affected the ΔE (P<0.0001). The minimum veneering porcelain thickness for the shade match was 2 mm for NC and 1.8 mm for AM.

***Conclusion***. Veneering porcelain thickness and background shade affected the shade match of zirconia-based restorations. With dark-shaded backgrounds, the amount of veneering porcelain thickness needed for the shade match might be beyond acceptable clinical limits. Tooth-shaded backgrounds are esthetically advocated rather than dark-shaded backgrounds in zirconia-based restorations.

## Introduction

Zirconia-based restorations are an acceptable treatment option in restorative dentistry because of their appropriate strength and esthetics.^1-3^ Zirconia-based restorations have two components, including a zirconia coping and a veneering porcelain. Zirconia copings represent benefits of high fracture strength,^4^ proper optical properties^5^ and a white-to-ivory color.^6^ Veneering porcelains create a tooth-like appearance due to their proper shade, translucency and tooth-like appearance.^7^ Consequently, zirconia-based restorations have become popular in dentistry.^8^

In order to quantify the color of an object, different color systems have been developed in color science. One of the most commonly applied color systems in dentistry is CIELab, in which L^*^, a^*^ and b^*^ denote lightness, redness-greenness, and yellowness-blueness, respectively.^9,10^ Additionally, the CIELab is used to determine the color difference between two objects. A single value, known as ∆E, is calculated from the formula: ΔE^*^_ab_= [(L^*^_2_- L^*^_1_)^2^+ (a^*^_2_- a ^*^_1_)^2^+ (b^*^_2_- b^*^_1_)^2^]^1/2^ in order to measure the color difference.^9,10^ Thereafter, the ∆E is compared with acceptability and perceptibility thresholds in order to evaluate the visibility of the color difference to human eyes.^11-14^ The CIELab system has been reasonably employed to determine the shade reproduction of different ceramics and restorations.^15-18^

Zirconia ceramics are optically semi-translucent materials.^9,10^ Depending on the shade, brand and thickness, zirconia ceramics exhibit different absolute translucencies (visible light transmittance percentage) from low (20%) to ultra (49%) levels. ^9,19,20^ Therefore, zirconia might manifest the color of its underlying materials such as backgrounds and luting agents, leading to an improper shade for zirconia-based restorations.^9^ A 1-mm-thick zirconia ceramic is needed to create an acceptable masking ability regardless of background color.^21^ The background L^*^ value affects the masking ability of an 0.5-mm-thick white zirconia ceramic.^22^ A zirconia-based restoration commonly includes a 0.3- to 0.5-mm-thick zirconia coping and a 1-mm-thick veneering porcelain. Although an 0.4-mm-thick zirconia coping alone produces acceptable masking ability on tooth-colored backgrounds, it might be insufficient for masking metal backgrounds.^23^ Zirconia-based restorations with an 0.4-mm-thick coping might not be color-matched with natural teeth when placed on gold alloy posts and cores.^24^ An 0.4-mm-thick zirconia coping alone represents a relative masking ability on color backgrounds, which might lead to color mismatches;^24^ however, when this zirconia coping is layered with an 0.4-mm-thick veneering porcelain, the resultant color might be further adjusted.^25^ Moreover, the shade of zirconia-based restorations on a dark background might depend on veneering thickness.^26^ Some color mismatches have been reported at the cervical area of zirconia-based restorations with 0.3 and 0.5-mm-thick copings.^27^ Controversially, no significant differences have been reported between CIELab values of zirconia-based crowns on metal backgrounds and composite resin backgrounds.^28^

Various backgrounds and veneering porcelain thicknesses might be used in zirconia-based restorations, inducing different color results. However, according to the controversial results derived from the literature, effects of veneering porcelain thickness and background shade on the shade match of zirconia-based restorations are not clearly understood. Therefore, the aim of this in vitro study was to evaluate the effects of veneering porcelain thickness and background shade on the shade match of zirconia-based restorations. The null hypothesis was that the veneering porcelain thickness and background shade would not affect the shade match of zirconia-based restorations.

## Methods

A sample size of n=10 was determined in each study group by considering α=0.05, β=0.1, the study design and previous studies.^21,23^ Thus, 40 veneered zirconia disk specimens with 0.4 mm zirconia thickness and 4 veneering porcelain thicknesses (1.6, 1.8, 2.0, and 2.2 mm) were fabricated. The range of veneering porcelain thickness was selected based on a pilot study.

A computer-aided design/computer-aided manufacturing system (CORiTEC 250i, imes-icore GmbH, Eiterfeld, Germany) was employed to mill zirconia blanks (VITA YZ T, VITA Zahnfabrik H. Rauter GmbH & Co. KG, Bad Säckingen, Germany) and to prepare zirconia disks. The zirconia disks were 0.4 mm in thickness and 10 mm in diameter. The zirconia disks were shaded with an A2 shade coloring liquid (Medium YZ T Coloring Liquid, VITA Zahnfabrik H. Rauter GmbH & Co. KG, Bad Säckingen, Germany) through a 2-minute-immersion process. The zirconia disks were sintered at 1480°C through a 12-hour process in a sintering furnace (iSINT HT, imes-icore GmbH, Eiterfeld, Germany) and were then adjusted to achieve the intended thickness of 0.4±0.02 mm using a zirconia polishing kit (BruxZir, Glidewell Direct, Irvine, CA, USA). The zirconia disk was eliminated from the study in case of lack of the intended thickness. Finally, the zirconia disks were cleaned in a 98% ethanol solution and air-dried.

An A2 shade feldspathic veneering ceramic for zirconia frameworks (VITA VM9, VITA Zahnfabrik H. Rauter GmbH & Co. KG, Bad Säckingen, Germany) was used to veneer zirconia disks with regard to intended thicknesses using manual add-on technique. The veneered zirconia disks were fired for 69 minutes at a heat rate of 55°C/min from 500°C to 910°C, cooled to room temperature, and polished by using a porcelain polishing laboratory kit (All Ceramic Extra-Oral Kit, Cosmedent Inc., Chicago, IL, USA). A 3-step polishing procedure was performed using green, purple and yellow polishing disks, as instructed by the manufacturer. The veneered zirconia disks were adjusted to achieve the intended thicknesses (±0.02) using the same porcelain polishing/adjusting kit. The veneered zirconia disk was eliminated from the study in case of lack of the intended thickness. The veneered specimens were cleaned and dried with the aforementioned methods.

**Figure 1:**
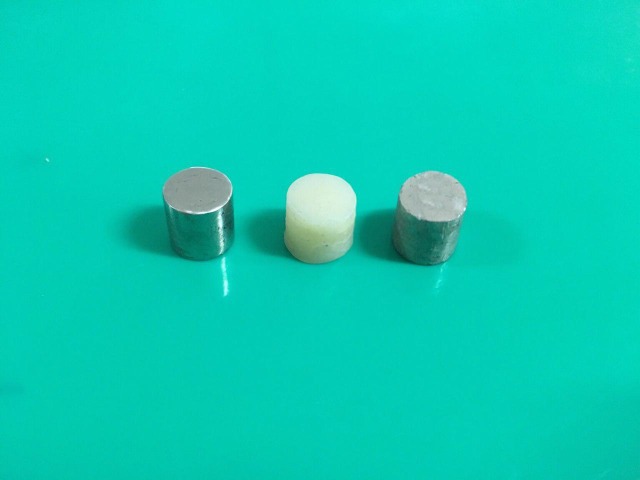
Backgrounds tested (from left to right: nickel‒chromium, A2 shade composite resin, amalgam).

Three cylindrical backgrounds were made from an A2 shade light-polymerized composite resin (Z100 Restorative, 3M ESPE, St. Paul, MN, USA), a nickel‒chromium alloy (VeraBond V, Alba Dent, Fairfield, CA, USA), an amalgam alloy (Dispersalloy Dispersed Phase Alloy Regular Set 3 Spill (800 mg) Yellow Caps, Densply Sirona, York, PA, USA). The composite resin was applied to a plastic mold and incrementally polymerized with a light-polymerizing unit (Elipar FreeLight 2, 3M ESPE, St. Paul, MN, USA) for 40 seconds with an intensity of 800 mW/cm^2^ to fabricate the composite resin background (A2). A wax pattern was cast to prepare the nickel‒chromium background (NC). The amalgam alloy was triturated and condensed in a plastic mold to fabricate the amalgam background (AM). All the backgrounds were 10 mm in diameter and 10 mm in height (Figure 1).^21,22^ CIELab values of the backgrounds were measured with a spectrophotometer^29^ (SpectroShade Micro, MHT Optic Research AG, Verona, Italy) (A2: L^*^=63.4, a^*^=0.5, b^*^=18.4; NC: L^*^=11.9, a^*^= -1.1, b^*^=1.5; AM: L^*^=25.4, a^*^= -0.5, b^*^=4.9).

**Figure 2:**
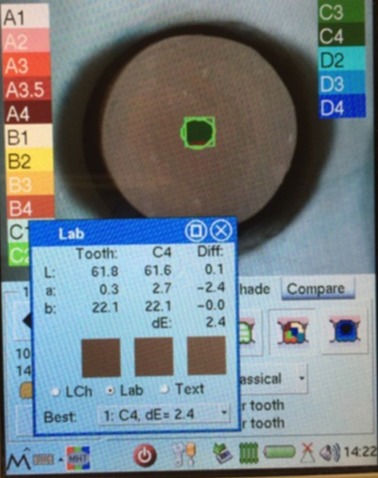
The spectrophotometer’s screen measuring the CIELab values of a specimen.

The same spectrophotometer and a customized putty mold were employed for color measurments.^15,21,23^ This mold prevented external lights, supplied a seat for the backgrounds and matched the conditions of spectrophotometry for all the specimens.^17,24^ The specimens were seated on the backgrounds without an intermediate.^23,25^ Then the CIELab color measurements were performed by an expert operator at the center of the specimens 3 times for each specimen and the average values were recorded (Figure 2). Also the CIELab values were measured for a new A2 VITA classical shade tab (target shade) at the center of its middle third (L^*^=74.8, a^*^=0.7, b^*^=20.0).^30^ The spectrophotometer confirmed the A2 shade for the tab. In order to determine the color difference between the specimens and the target shade, ∆E was calculated from this formula: ΔE^*^_ab_= [(ΔL^*^)^2^+ (Δa^*^)^2^+ (Δb^*^)^2^]^1/2^. An acceptability threshold of ΔE=3.7^11^ was assumed to assess the color differences between the specimens and the target shade and to judge the specimens’ shade match.

Data were analyzed with SPSS 21 (SPSS Inc., Chicago, IL, USA). Kolmogorov-Smirnov test indicated the normal distribution of data in all the studied groups (P>0.05). The effects of veneering porcelain thickness, background shade and their interaction on the CIELab and ∆E values were evaluated using repeated-measures ANOVA. Pairwise comparisons of the studied groups were performed using the Bonferroni correction. The ΔE values of the studied groups were compared with the threshold for acceptability (ΔE=3.7) with STATA (StataCorp LP, Lakeway, TX, USA) using one-sample t-test. The 0.05 level of significance was considered for all the tests.

## Results

**Figure 3:**
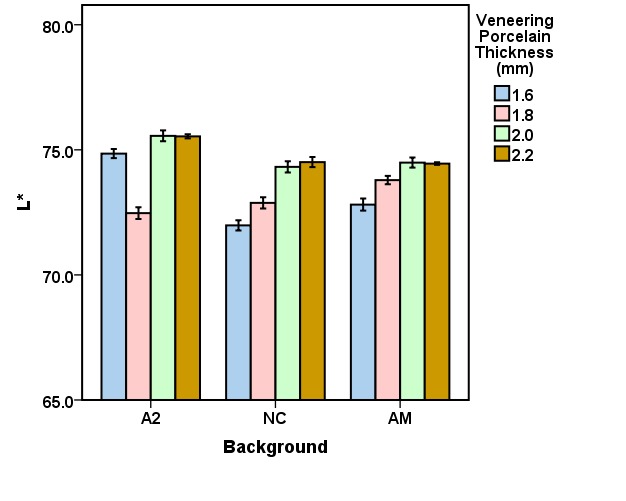
Means and standard deviations of lightness (L^*^) values.

**Figure 4:**
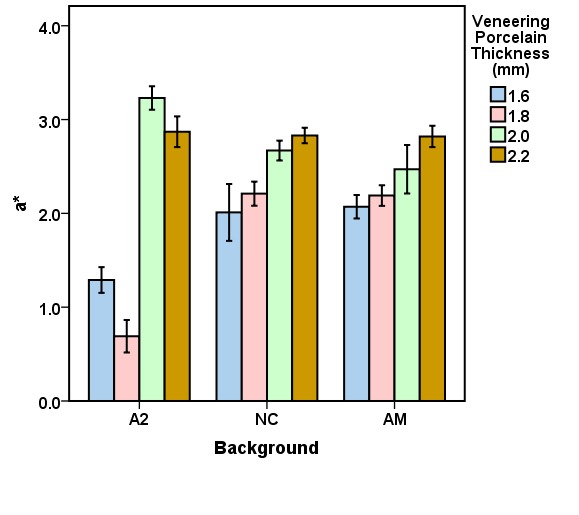
Means and standard deviations of red-green (a^*^) values.

**Figure 5:**
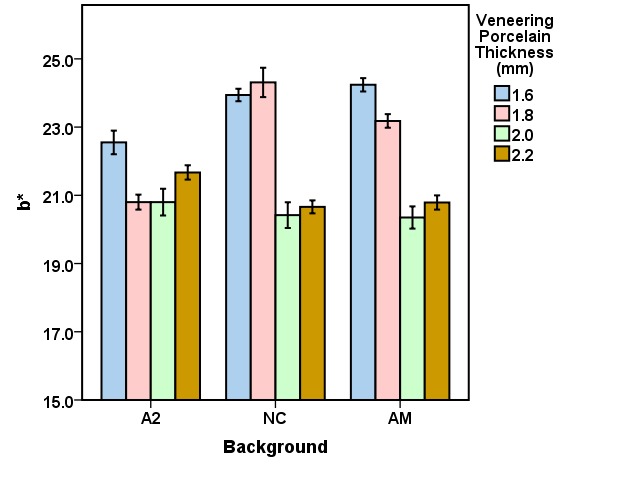
Means and standard deviations of yellow-blue (b^*^) values.

**Figure 6:**
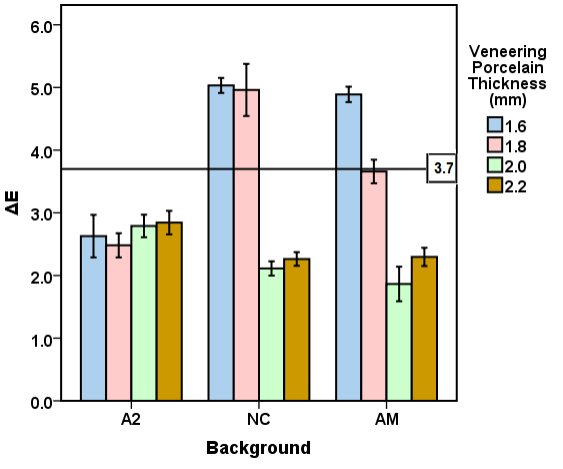
Means and standard deviations of color difference (∆E) values.

The mean CIELab and ΔE values for the veneering porcelain thicknesses of 1.6, 1.8, 2.0, and 2.2 mm for the studied backgrounds (A2, NC, AM) are presented in Figures 3 to 6. Repeated-measures ANOVA results indicated that the veneering porcelain thickness (P<0.0001), the background shade (P<0.0001) and their interaction (P<0.0001) significantly affected the CIELab and ΔE values (Table 1). Pairwise comparisons of the ΔE values using the Bonferroni correction indicated significant differences between the thickness groups for each background (P<0.05) and between the backgrounds for each thickness group (P<0.05).

**Table 1:** Results of repeated measures ANOVA (Greenhouse-Geisser) on effects of veneering porcelain thickness and background shade on color attributes

**Color attributes**	**Source**	**Type III Sum of Squares**	**df**	**Mean Square**	**F**	**p**
**L** ^*^	Background	28.408	2	14.204	405.724	<0.0001
	Thickness	85.285	3	28.428	730.376	<0.0001
	Background × Thickness	40.892	6	6.815	184.953	<0.0001
	Error	1.990	54	0.037		
**a** ^*^	Background	4.066	2	2.033	66.276	<0.0001
	Thickness	34.622	3	11.541	455.834	<0.0001
	Background × Thickness	18.023	6	3.004	114.294	<0.0001
	Error	1.419	54	0.026		
**b** ^*^	Background	17.017	2	8.509	67.090	<0.0001
	Thickness	185.051	3	61.684	875.827	<0.0001
	Background × Thickness	70.659	2.946	23.981	158.146	<0.0001
	Error	4.021	26.518	0.152		
**∆E**	Background	16.480	2	8.240	170.256	<0.0001
	Thickness	79.102	3	26.367	490.950	<0.0001
	Background × Thickness	57.409	6	9.568	191.446	<0.0001
	Error	2.699	54	0.050		

One-sample t-test was used to compare the means of ΔE values with the acceptability threshold (ΔE=3.7). The null hypothesis of ΔE≤3.7 was rejected for NC in 1.6 (P<0.0001) and 1.8 (P<0.0001), and for AM in 1.6 (P<0.0001), while it was not rejected for NC in 2.0 (P=1) and 2.2 (P=1), for AM in 1.8 (P=0.744), 2.0 (P=1), and 2.2 (P=1), and for A2 in all thickness groups (P=1).

## Discussion

According to the results of this study, which indicated significant differences in the CIELab and ΔE values in relation to veneering porcelain thickness and background shade, the null hypothesis of the study was rejected. With 0.4-mm zirconia coping, the minimum porcelain thickness for the shade match was 2 mm for NC and 1.8 for AM, while all the tested thicknesses resulted in shade match for A2.

The results are interpreted in consideration of the absolute translucency of zirconia and the color of the backgrounds. Zirconia is semi-translucent and has an absolute translucency between 20% and 49% in a thickness of 1 mm based on the zirconia shade and brand.^9^ Veneering porcelains are more translucent than zirconia.^9^ Thus, a background might express its color under the zirconia coping, affecting the resultant color of a zirconia-based ceramic. The resultant color is the outcome of the ceramic and background colors. Since the backgrounds exhibited different CIELab values, they affected the resultant color in different degrees. The background color initiates the difference in the minimum veneering porcelain thickness needed for esthetics. A greater color difference between the background and the target shade needed a greater veneering porcelain thickness to gain a proper shade match. This is why the general ranking for the proper veneering porcelain thickness needed for the backgrounds was NC>AM>A2 (Figure 6). The thickness needed for NC was the highest because of its greatest color differences compared with the target. As the ceramic thickness increases, the ceramic translucency and the background effect decrease.^7,9,19,21,30^ That is why the increase in the veneering porcelain thickness from 1.6 to 1.8 mm for AM and from 1.6 to 2 mm for NC resulted in an appropriate shade match (Figure 6).

Barizon et al,^7^ Wang et al,^19^ and Choi and Razzoog^25^ in separate studies showed the crucial effect of ceramic thickness on translucency and masking ability of glass ceramic and zirconia restorations. They reported that increasing the ceramic thickness decreased the ceramic translucency and increased the ceramic masking ability. The present study confirmed the results of these studies and additionally introduced proper veneering porcelain thicknesses for zirconia-based restorations on dark backgrounds for gaining a shade match.

Suputtamongkol et al^28^ evaluated the effect of the color of background on the final color of zirconia-based crowns and reported no significant differences between the color of zirconia-based crowns with increased thicknesses (2.3 mm) on metal backgrounds and composite resin backgrounds. Their result on the background’s effect was consistent with the present study. Both studies showed that increasing the total restoration thickness beyond the clinically recommended restoration thickness (1.5 mm) led to the color match in zirconia-based restorations regardless of the background color.

Tabatabaian et al^23^ assessed the effect of zirconia coping thickness and background type on the color masking ability of zirconia-based restorations. They reported that the thickness of zirconia coping should be at least 0.8 mm in order to attain an ideal masking ability on nickel-chromium alloy, while it could be 0.4 mm for tooth-colored backgrounds. However, the present study remarked the veneering ceramic thickness for shade matching. Since the translucency of feldspathic ceramics is more than that of zirconia ceramics, greater thickness for feldspathic ceramics than for zirconia ceramics is needed to achieve an ideal masking ability. However, feldspathic ceramics are optically more similar to tooth structures of dentin and enamel than zirconia ceramics.^9^ Therefore, the shade/translucency matching of zirconia-based restorations seems more achievable with increasing the veneering ceramic thickness than with increasing the zirconia coping thickness. However, both studies confirmed an increase in the restoration thickness with dark-shaded backgrounds for esthetics.

Oh and Kim^24^ reported that background shade (gold alloy; base metal alloy; A1, A2, A3, and A4 composite resins), total ceramic thickness (1 and 1.5 mm), and zirconia coping brand (Lava, Cercon, Zirkonzahn) affected the resultant color of zirconia-based restorations with 0.4-mm-thick zirconia coping layered with IPS e.max Press Ceram. They showed that only Lava crowns on gold alloy cores could not create a color match, because their ∆E color difference values were more than the acceptability threshold (close to 5.5). The discrepancies in the results of the studies on the effects of ceramic thickness and background shade might be attributed to the difference in zirconia coping and veneering ceramic brands, control groups, and acceptability thresholds used by the studies.

According to the results of this study, increasing the veneering porcelain thickness of zirconia-based restorations improves the esthetic outcomes; however, this might increase the risk of porcelain chipping.^31,32^ Therefore, with consideration of both esthetic and mechanical properties, the use of tooth-shaded backgrounds rather than dark metal backgrounds is recommended for zirconia-based restorations. An increase in zirconia coping thickness might be a solution for the esthetics in zirconia-based restorations, which was not evaluated in this study. The assessment of the effect of zirconia coping thickness on the color match of zirconia-based restorations is suggested for future research studies.

The color of zirconia-based restorations might be affected by factors such as background, luting agent, zirconia coping (thickness, translucency, shade, brand), veneering ceramic (thickness, translucency, shade, brand), glaze, surface staining, and laboratory procedures (coloring, sintering and firing conditions, polishing and finishing).^9^ Among these factors, veneering ceramic thickness and background shade were investigated in this study and accordingly, using a background with a shade corresponding to the target shade is advised instead of dark-shaded backgrounds (amalgam and nickel‒chromium alloys) for zirconia-based restorations. In case of a dark-shaded background, increasing the restoration thickness might be considered. However, an increase in zirconia coping thickness might be more advantageous than an increase in veneering porcelain thickness alone regarding both mechanical and esthetic properties.

This study did not evaluate some affective factors such as luting agent, thickness and brand of zirconia coping, brand of dentin veneering ceramic, enamel veneering ceramic, glaze, veneering technique, and external staining. Therefore, evaluation of these factors is suggested for future investigations.

## Conclusion

Within the limitations of this in vitro study, the following conclusions were drawn:

1. Veneering porcelain thickness and background shade affected the shade match of zirconia-based restorations.
2. With dark-shaded backgrounds, the amount of veneering porcelain thickness needed for the shade match might be beyond the acceptable clinical limits.
3. In order to create the shade match for zirconia-based restorations, tooth-shaded backgrounds are advocated rather than dark-shaded backgrounds.

## Conflict of Interests

The authors declare no conflict(s) of interest related to the publication of this work.

## Authors’ contributions

FT contributed to the concept and design of the work. The acquisition, analysis, and interpretation of data were accomplished by FT, KA, and MN. FT, KA, and MN drafted and revised the manuscript critically for intellectual content. All the authors read and approved the final manuscript.

## Funding

This study was supported by a grant from the Research Deputy of School of Dentistry of Shahid Beheshti University of Medical Sciences, Tehran, Iran [grant number 11325].

## Acknowledgments

The authors thank the Research Deputy of School of Dentistry of Shahid Beheshti University of Medical Sciences for the financial support (the grant No. 11325), Mehr Dental Lab for their CAD/CAM support, and Mr Aliakbar Deghost for his laboratory work in preparing specimens.

## Ethics approval

Not applicable.
